# Chamaejasmine Isolated from *Wikstroemia dolichantha* Diels Suppresses 2,4-Dinitrofluoro-benzene-Induced Atopic Dermatitis in SKH-1 Hairless Mice

**DOI:** 10.3390/biom9110697

**Published:** 2019-11-05

**Authors:** Tae-Young Kim, No-June Park, Jonghwan Jegal, Sangho Choi, Sang Woo Lee, Jin Hang, Su-Nam Kim, Min Hye Yang

**Affiliations:** 1 College of Pharmacy, Pusan National University, Busan 46241, Korea; taeyour@pusan.ac.kr (T.-Y.K.); jhjegal@pusan.ac.kr (J.J.); 2 Natural Products Research Institute, Korea Institute of Science and Technology, Gangneung 25451, Korea; 115519@kist.re.kr; 3 International Biological Material Research Center, Korea Research Institute of Bioscience and Biotechnology, Daejeon 34141, Korea; decoy0@kribb.re.kr (S.C.); ethnolee@kribb.re.kr (S.W.L.); 4 Institute of Medicinal Plants, Yunnan Academy of Agricultural Sciences, Kunming 650205, China; jinhang1516@sina.com

**Keywords:** chamaejasmine, *Wikstroemia dolichantha*, 2,4-dinitrochlorobenzene, atopic dermatitis, skin barrier function, interleukin 4

## Abstract

Plants of the genus *Wikstroemia* have long been used as traditional medicines to treat diseases like pneumonia, rheumatism, and bronchitis. This study was designed to determine the effect of chamaejasmine, a biflavonoid present in *W. dolichantha*, on atopic dermatitis (AD)-like skin lesions in a 2,4-dinitrochlorobenzene (DNCB)-induced murine model of AD. Initially, we examined the anti-allergic activities of ten flavonoids from *W. dolichantha* by measuring β-hexosaminidase release from RBL-2H3 cells. Subsequently, an SKH-1 hairless mouse model of AD was developed based on the topical application of DNCB. Chamaejasmine (0.5%) or pimecrolimus (1%, positive control) were applied to dorsal skins of DNCB-sensitized AD mice for two weeks. Serum IL-4 and IgE levels were determined using enzyme-linked immunosorbent assay kits and transepidermal water loss (TEWL) and skin hydration were measured using a Tewameter TM210 and a SKIN-O-MAT, respectively. Of the ten flavonoids isolated from *W. dolichantha*, chamaejasmine most potently inhibited DNP-specific IgE-induced degranulation in RBL-2H3 cells. Topical administration of chamaejasmine attenuated the clinical symptoms of DNCB-induced dermatitis (i.e., itching, dryness, erythema, and edema). Histological analyses demonstrated that dermal thickness and mast cell infiltration in dermis were significantly reduced by chamaejasmine. In addition, 0.5% chamaejasmine inhibited DNCB-induced increases in total IL-4 and IgE levels in serum, improved skin barrier function, and increased epidermis moisture. Our findings suggest chamaejasmine might be an effective therapeutic agent for the treatment of atopic diseases.

## 1. Introduction

The atopic dermatitis (AD) is a chronic skin disorder provoked by immune system disturbance and is characterized by itching, redness, skin keratinization, and exudates. AD is caused by a complex combination of genetic, environmental, physiological and pharmacological factors and is also associated with other allergic diseases such as allergic rhinitis, asthma, and conjunctivitis [1,2]. The impact of AD on health-related quality of life is considerable, and in addition, this skin disease causes a significant burden in terms of increased health care utilization and costs [3]. AD can be classified as extrinsic and intrinsic AD depending on whether immunoglobulin E (IgE) specific to an external allergen is involved; IgE-associated AD (extrinsic AD) is the classical type of this disease and has a high prevalence, whereas the prevalence of non IgE-mediated AD (intrinsic AD) is approximately 20% [4]. The known functions of IgE and mast cells in allergic inflammation suggest that IgE-mediated mast cells play major effector roles in the pathogenesis of AD [5,6]. For this reason, blocking the productions of high levels of Th2 cytokines (e.g., IL-4) and enzymes (e.g., β-hexosaminidase, an enzyme found in preformed mast cell granules) by mast cells may provide a strategy for treating AD [1,6].

*Wikstroemia dolichantha* Diels (Thymelaeaceae) is a perennial shrub widely distributed in southern China and is commonly used as an herbal medicine [7]. In China, *Wikstroemia* species have been used in folk medicines to treat hepatitis and arthritis [7,8]. The aerial parts of *Wikstroemia* species have been reported to contain predominantly flavonoids, biflavonoids, and coumarins [9,10]. Furthermore, research supports the diverse pharmacological actions and potential clinical applications of *Wikstroemia* species as anti-bacterial, anti-viral, anti-tumor, and antifertility agents [11,12]. Previously, we found that an ethanolic extract of the aerial parts of *W. dolichantha* strongly inhibited β-hexosaminidase release from RBL-2H3 cells in vitro and exhibited anti-dermatitis activities on the atopic dermatitis (AD)-like skin lesions in an animal model [13]. In the present study, we undertook isolation of the bioactive components responsible for the anti-inflammatory and anti-atopic activities of *W. dolichantha* extract. Accordingly, we investigated the suppressive effects of ten flavonoids from isolated *W. dolichantha* extract on β-hexosaminidase release from IgE-stimulated RBL-2H3 cells and on dermatitis in a 2,4-dinitrochlorobenzene (DNCB)-induced AD murine model of AD.

## 2. Materials and Methods

### 2.1. Equipment Used

^1^H- and ^13^C-NMR, COSY, HSQC, HMBC, and NOESY data were obtained using a superconducting FT-NMR 400 or 500 MHz spectrometer (Agilent Technologies, Santa Clara, CA, USA). HR-ESI mass spectra were recorded on an Agilent Technologies, 6530 Accurate-Mass Q-TOF LC/MS. The HPLC system (Shimadzu, Tokyo, Japan) consisted of a UV/VIS detector (model SPD-20A), two pumps (model LC-20AT), a system controller (model CBM-20A) and a workstation (model HW-2000 solution). Column chromatography was performed using Sephadex LH-20 gel (25–100 μM mesh, Pharmacia, Stockholm, Sweden) and silica gel (230–400 mesh, Merck, Darmstadt, Germany).

### 2.2. Plant Material and Extraction 

The aerial parts of *Wikstroemia dolichantha* Diels were collected in Yunnan Province, Lijiang, China and identified by Dr. Sang Woo Lee (Korea Research Institute of Bioscience and Biotechnology). A voucher specimen (PNU-0024) was deposited at the Medicinal Herb Garden, Pusan National University. Dried aerial parts of *W. dolichanta* (5 kg) were extracted with 95% EtOH (12 L × 3) and evaporated under reduced pressure to yield *W. dolichanta* EtOH extract (WDE) (466.8 g, 9.3% extract yield).

### 2.3. Compound Isolation 

WDE was suspended in distilled water (1.6 L) and successively partitioned with *n*-hexane (4.8 L), EtOAc (4.5 L), and *n*-BuOH (5.5 L). The active EtOAc soluble fraction (39.9 g) was chromatographed on a silica gel column using CH_2_Cl_2_-MeOH (20:1 → 100% MeOH, gradient system) as eluent to yield ten fractions (WDE1~WDE12). Fraction WDE3 (492.0 mg) was fractionated into five subfractions (WDE3-1~WDE3-5) by Sephadex LH-20 column chromatography using MeOH, and subfraction WDE3-3 (74.9 mg) was subjected to Silica gel isocratic CC (column chromatography) using CH_2_Cl_2_-MeOH (40:1) as eluent to yield four fractions (WDE3-3-1~WDE3-3-4). Subfraction WDE3-3-4 (63.9 mg) was fractionated into two subfractions (WDE3-3-4-1~WDE3-3-4-2) by Sephadex LH-20 column chromatography using MeOH. Compound **1** [padmatin (12.3 mg)] was obtained as pure powder by filtering fraction WDE3-3-4-1. Fraction WDE4 (492.0 mg) was fractionated into five subfractions (WDE4-1~WDE4-5) by Silica gel CC using EtOAc-Hex (5:1 → 100% EtOAc, gradient system). Subfraction WDE4-3 (84 mg) was suspended in MeOH, and compound **2** [aromadendrin (35.1 mg)] was obtained by filtration. The resulting filtrate of WDE4-3 was then fractionated into five subfractions (WDE4-3-1~WDE4-3-5) by Sephadex LH-20 using MeOH, and compound **3** [apigenin (5.6 mg)] was obtained as pure powder by filtering fraction WDE4-3-4. Subfraction WDE4-4 (62.9 mg) was fractionated into five subfractions (WDE4-4-1~WDE4-4-5) by Sephadex LH-20 using MeOH, and subfraction WDE4-4-4 (16.3 mg) was subjected to RP HPLC (Watchers 120 ODS-BP, S-10 μm, 150 × 10 mm; detection, UV at 254 nm; flow rate, 2 mL/min) and eluted with a AcCN-H_2_O isocratic system (5:5, 50 min) to yield compound **4** [wikstaiwanone C (3.5 mg, *t*_R_ 36 min)]. Fraction WDE6 (4.7 g) was chromatographed on a Silica gel CC using CH_2_Cl_2_-MeOH (10:1 → 100% MeOH, gradient system) as eluent to yield five fractions (WDE6-1~WDE6-5). Compound **5** [taxifolin (35.1 mg)] was obtained as a pure powder by recrystallizing the filtrate of subfraction WDE6-3 from MeOH. Subfraction WDE6-2 (467.4 mg) was fractionated into six subfriactions (WDE6-2-1~WDE6-2-6) by Sephadex LH-20 using MeOH, and compounds **6** [neochamaejasmine B (3.3 mg)], **7** [chamaejasmine (93 mg)], and **8** [naringenin (88.5 mg)] were obtained as pure powders by filtering fractions WDE6-2-3, WDE6-2-5, and WDE6-2-6, respectively. Fraction WDE7 (5.0 g) was subjected to silica gel CC using CH_2_Cl_2_-MeOH (10:1 → 100% MeOH, gradient system) as eluent to yield three subfractions (WDE7-1~WDE7-3). Subfraction WDE7-3 (2.0 g) was subjected to Silica gel CC using CH_2_Cl_2_-MeOH (8:1 → 100% MeOH, gradient system) to yield five fractions (WDE7-3-1~WDE7-3-5), and compound **9** [afzelechin (102.3 mg)] was obtained as a pure powder by filtering fraction WDE7-3-1. Fraction WDE8 (2.6 g) was subjected to Silica gel CC using CH_2_Cl_2_-MeOH (20:1 → 100% MeOH, gradient system) as eluent to yield five fractions (WDE8-1~WDE8-5). Subfraction WDE8-3 (149.6 mg) was fractionated into five subfractions (WDE8-3-1~WDE8-3-5) by Sephadex LH-20 (MeOH), and subfraction WDE8-3-3(71.1mg) was subjected to silica gel CC using CH_2_Cl_2_-MeOH (10:1 → 100% MeOH, gradient system) as eluent to yield two fractions (WDE8-3-3-1~WDE8-3-3-2). Compound **10** [catechin (38.2 mg)] was obtained as pure powder by filtering fraction WDE8-3-3-2.

### 2.4. RBL-2H3 Cell Culture 

The rat basophilic leukemia cell line, RBL-2H3, was purchased from the American Type Culture Collection (CRL-2256, Bethesda, MD, USA). Cells were grown in minimum essential medium (MEM) containing Eagle’s salts, 10% fetal bovine serum (FBS), 2 mM L-glutamine, 100 U/mL penicillin, and 100 μg/mL streptomycin in a humidified 5% CO_2_/air atmosphere at 37 °C.

2.5. β-Hexosaminidase Release Assay

RBL-2H3 cells were treated overnight with anti-dinitrophenyl immunoglobulin E (anti-DNP IgE), rinsed out with Siraganian buffer, and incubated in buffer for 10 min. Cells were then treated with DMSO or separately with each of the ten isolates of *W. dolichantha* (30 μM) for 1 h and sensitized with DNP-BSA antigen (10 μg/mL) for 20 min to provoke degranulation. Supernatants were transferred to 96-well plates and incubated with 1 mM of 4-nitrophenyl-*N*-acetyl-β-d-glucosaminide as substrate in 0.1 M citrate buffer for 3 h at 37 °C. Absorbances were measured using a microplate reader at 405 nm.

### 2.6. Animals 

Six-week-old female SKH-1 hairless mice purchased from Orient Bio Inc. (Seongnam, Republic of Korea) were housed in an air-controlled animal room (25 ± 5 °C, 55 ± 5% RH). Animals were fed standard laboratory food and water *ad libitum*. All animal experiments were conducted in accord with the Guide for the Care and Use of Laboratory Animas issued by the National Institute of Health (NIH publication No. 85-23, revised 2011) and authorized by the Institutional Animal Care and Use Committee of KIST (Certification No. KIST-2016-011).

### 2.7. Induction of DNCB-Induced AD and Treatment with Chamaejamine from W. dolichantha 

2,4-Dinitrochlorobenzene (DNCB; 1%) (Sigma-Aldrich, Seoul, Republic of Korea) in propylene glycol:EtOH (7:3 *v*/*v*) was used to induce AD-like symptoms. First, mice in the DNCB control group and the DNCB/isolated compound groups were sensitized by applying 1% DNCB in propylene glycol:EtOH (7:3 *v*/*v*) to dorsal skin once daily for a week (experimental day 1 (ED1) to ED7). DNCB controls were then challenged by administering 100 μL of 0.1% DNCB from ED8 every 3 days for 2 weeks. Animals were painted with 100 μL solution of 0.5% chamaejamine or 1% pimecrolimus, a positive control, in propylene glycol:EtOH (7:3 *v*/*v*) and with DNCB, as described above, twice a day from ED8 for 2 weeks. In these groups, chamaejamine or pimecrolimus were applied 4 h after DNCB application. Mice in the untreated control group were treated with propylene glycol:EtOH (7:3 *v*/*v*) daily using the method described above.

### 2.8. Histological Examination 

To examine histologic changes, dorsal skins were fixed in 10% formalin for 24 h and embedded in paraffin. Fixed tissues were sectioned at 2–3 mm, dried for 24 h at 37 °C, and stained with hematoxylin and eosin (H&E) or toluidine blue. Histological changes were observed under an optical microscope (Olympus CX31/BX51, Olympus Optical Co., Tokyo, Japan) and photographed (TE2000U, Nikon Instruments Inc., Melville, NY, USA).

### 2.9. Measurement of Transepidermal Water Loss (TEWL) and Skin Hydration 

Skin damage severity was evaluated by measuring transepidermal water loss (TEWL) and skin hydration, using a Tewameter TM210 (Courage and Khazaka, Cologne, Germany) and a SKIN-O-MAT (Cosmome, Rhur, Germany), according to the manufacturer’s instructions. TEWL and skin hydration were measured weekly under controlled conditions (25 ± 5 °C, 55 ± 5% RH).

### 2.10. Measurement of Total Serum IgE and IL-4 Levels 

Blood samples were obtained and centrifuged at 10,000 rpm for 15 min at 4 °C. Total serum IgE and IL-4 levels were measured using enzyme-linked immunosorbent assay kits (eBioscience, San Diego, CA, USA).

### 2.11. Statistical Analysis 

All quantitative data for this study were obtained through at least two independent experiments. In vitro data are shown as the means ± SDs and in vivo data are denoted as means ± SEMs for seven animals. Statistical analyses were carried out by a one-way analysis of variance (ANOVA) and a Tukey’s multiple comparisons post hoc analysis.

## 3. Results

### 3.1. Isolation of Flavonoids from W. dolichantha and Their Effects on β-Hexosaminidase Release by RBL-2H3 Cells 

**Figure 1:**
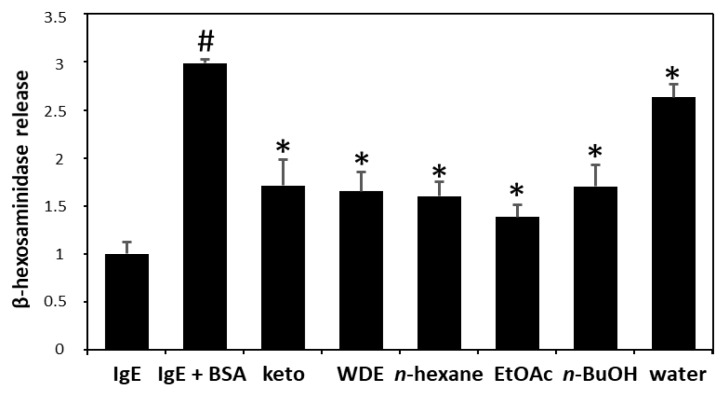
Anti-allergic effects of *Wikstroemia dolichantha* total extract (WDE) and fractions (*n*-hexane, EtOAc, *n*-BuOH, and water fraction) on β-hexosaminidase release from IgE-mediated RBL-2H3 cells. Results are expressed as the means ± SDs of two independent experiments. ^#^ *p* < 0.05 vs. vehicle control; * *p* < 0.05 vs. IgE + DNP-BSA treated cells. IgE = vehicle control, IgE + BSA = IgE + DNP-BSA treated cells, keto = 30 μM ketotifene.

**Figure 2:**
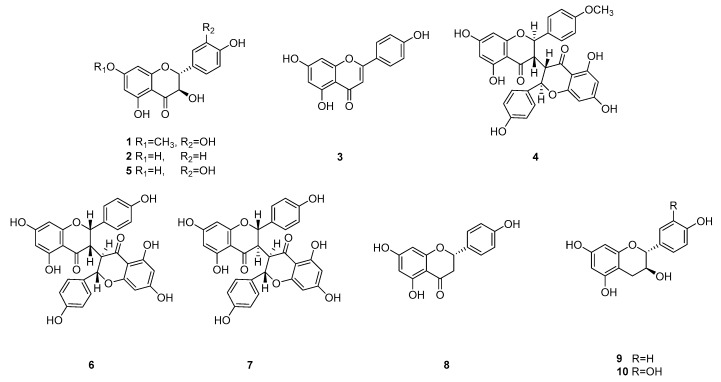
Structures of the ten flavonoids isolated from *Wikstroemia dolichantha* 95% ethanol extract. 1: padmatin, 2: aromadendrin, 3: apigenin, 4: wikstaiwanone C, 5: taxifolin, 6: neochamaejasmine B, 7: chamaejasmine, 8: naringenin, 9: afzelechin, 10: catechin.

The in vitro antiallergic and anti-inflammatory activity profiles of WDE and its extracts (*n*-hexane, EtOAc, *n*-BuOH, and water) were evaluated. The EtOAc extract was the most active and was not toxic to RBL-2H3 cells (Figure 1). This extract was subjected to silica gel column chromatography, Sephadex LH-20 column chromatography, and RP HPLC to yield ten known flavonoids, such as, padmatin (**1**) [14], aromadendrin (**2**) [15], apigenin (**3**) [16], (2*R*, 3*S*, 2′’*R*, 3′’*S*)-wikstaiwanone C (**4**) [17], taxifolin (**5**) [14], (2*R*, 3*R*, 2′’*R*, 3′’*S*)-neochamaejasmine B (**6**) [18], (2*S*, 3*R*, 2′’*S*, 3′’*R*)-chamaejasmine (**7**) [18], naringenin (**8**) [16], afzelechin (**9**) [19], and catechin (**10**) [20] (Figure 2). These ten flavonoids were identified using 2D-NMR and HR-MS data.

Error! Objects cannot be created from editing field codes.

**Figure 3:**
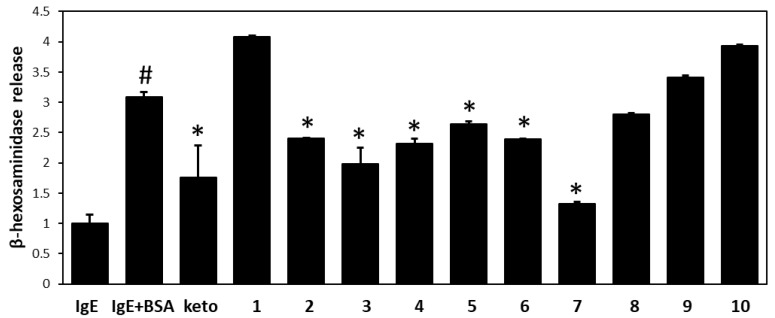
Anti-allergic effects of the ten flavonoids isolated from *W. dolichantha* on β-hexosaminidase release from IgE-mediated RBL-2H3 cells. Results are expressed as the means ± SDs of two independent experiments. ^#^ *p* < 0.05 vs. vehicle control; * *p* < 0.05 vs. IgE + DNP-BSA treated cells. IgE = vehicle control, IgE + BSA = IgE + DNP-BSA treated cells, keto = 30 μM ketotifene.

The antiallergic and anti-inflammatory effects of all flavonoids isolated from WDE were investigated by measuring β-hexosaminidase release from RBL-2H3 cells. This release was significantly greater from antigen-induced (anti-DNP IgE plus DNP-BSA) cells than from untreated controls (3.2-fold vs. untreated controls). Pretreatment with compounds **2**, **3**, **4**, **5**, **6**, and **7** at 30 μM effectively suppressed antigen-mediated β-hexosaminidase release from RBL-2H3 cells (Figure 3). In particular, **7** was most active against DNP-specific IgE-induced degranulation in RBL-2H3 cells.

### 3.2. Chamaejasmine (7) from W. dolichantha Ameliorated AD-like Skin Symptoms in DNCB-Induced Atopic Mice 

**Figure 4:**
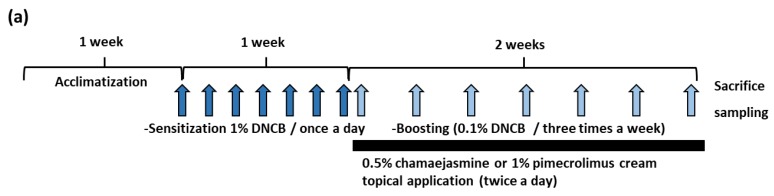

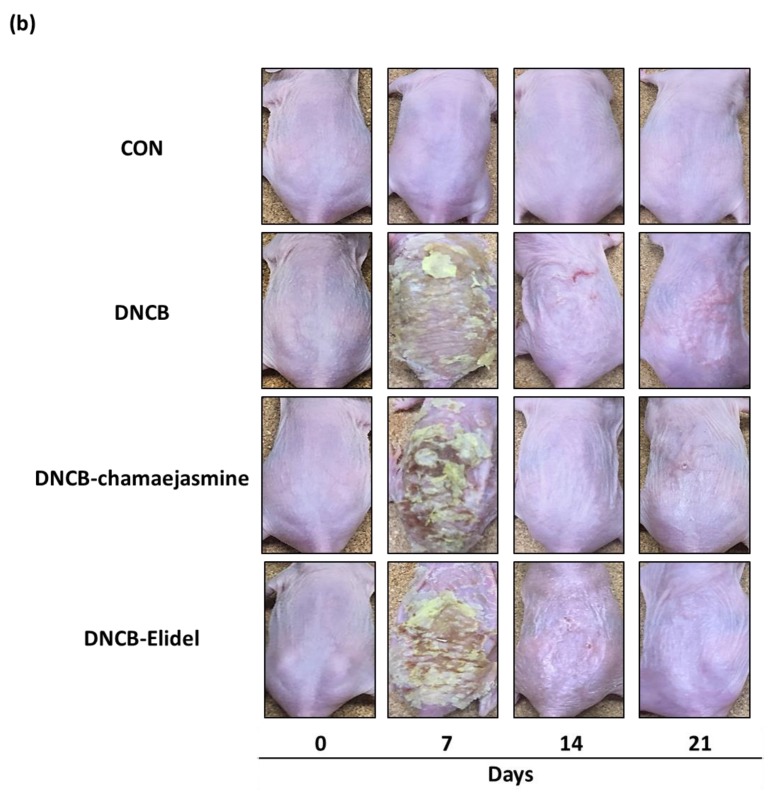
Effects of chamaejasmine on the development of DNCB-induced AD-like skin lesions in SKH-1 hairless mice. (**a**) Schematic representation of the experiment and (**b**) clinical features of DNCB-induced AD-like skin symptoms. CON: vehicle control group, DNCB: DNCB-treated control group, DNCB-chamaejasmine: DNCB and 0.5% chamaejasmine-treated group, DNCB-Elidel: DNCB and 1% pimecrolimus cream-treated group.

**Figure 5:**
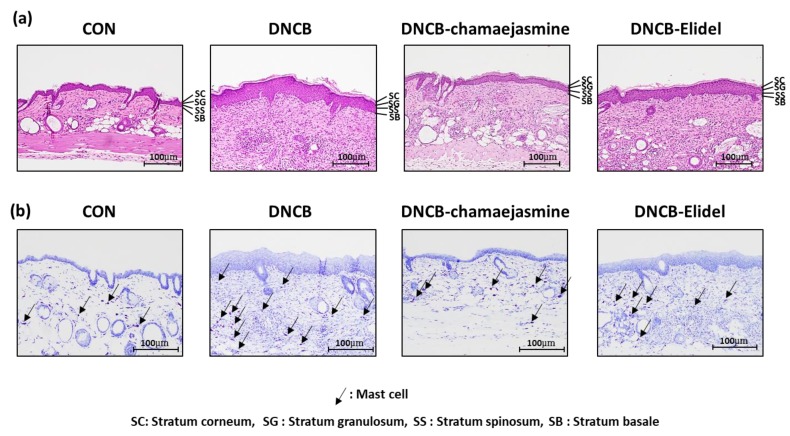

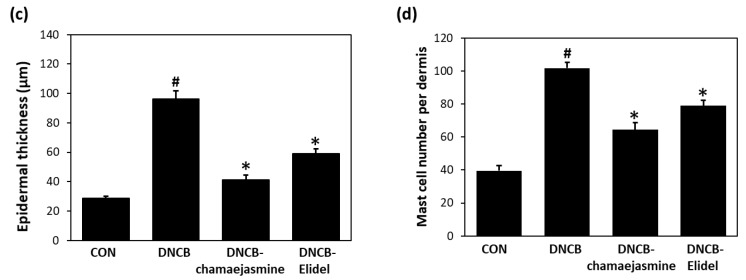
Effects of chamaejasmine on histopathological changes, epidermal thicknesses, and mast cell numbers in DNCB-induced AD-like skin lesions in SKH-1 hairless mice. (**a**) H&E staining, (**b**) toluidine blue staining, (**c**) epidermal thicknesses and (**d**) mast cell numbers. Results are expressed as the means ± SEMs of two independent experiments. ^#^ *P* < 0.05 vs. the CON group; * *P* < 0.05 vs. the DNCB group. CON: vehicle control group, DNCB: DNCB-treated control group, DNCB-chamaejasmine: DNCB and 0.5% chamaejasmine-treated group, DNCB-Elidel: DNCB and 1% pimecrolimus cream-treated group.

To investigate the effects of **7** on the skin lesions of DNCB-induced atopic mice, dermatitis levels were evaluated using skin lesion images. The procedure used to establish the DNCB-induced AD mouse model is shown in Figure 4a. On the last day of the experiment, DNCB application produced significant AD-like lesions, including erythema (with scratch marks), erosions, and dryness in the DNCB controls. Reduced AD-like symptom severity was observed in 0.1% DNCB-treated SKH-1 hairless mice co-treated with 0.5% **7** (DNCB-chamaejasmine group) (Figure 4b), in which the epidermal thickness of dorsal skin was 81% thinner than in the DNCB control group (Figure 5a,c). In addition, **7** application reduced the number of mast cells by 62% as compared with the DNCB controls group (Figure 5b,d). Pimecrolimus cream is a well-known treatment for atopic skin disorders and was used as a positive control. 1% Pimecrolimus cream reduced dorsal skin dermal thickness by 54% versus DNCB controls (Figure 5a,c) and mast cell infiltration by 33% (Figure 5b,d).

### 3.3. Chamaejasmine (7) Reduced Serum IgE and IL-4 Levels in DNCB-Induced Atopic Mice 

**Figure 6:**
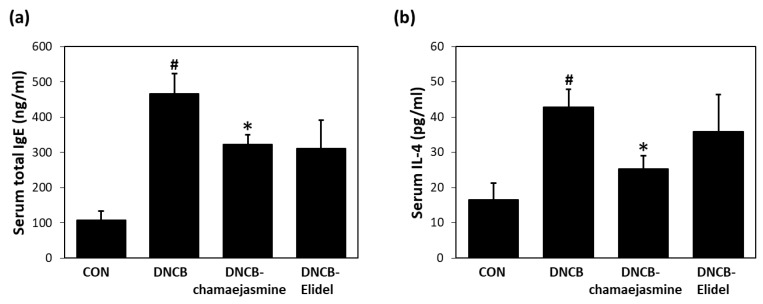
Effects of chamaejasmine on serum IgE and IL-4 levels in DNCB-sensitized SKH-1 hairless mice. (**a**) Serum total IgE levels, (**b**) serum total IL-4 levels. Serum samples were collected and tested for IgE and IL-4 concentrations on the last day of the experiment (experimental day 21). Results are expressed as the means ± SEMs (*n* = 7) of two independent experiments. ^#^ *P* < 0.05 vs. the CON group; * *P* < 0.05 vs. the DNCB group. EtOH extract of *Wikstroemia dolichantha*, CON: vehicle control group, DNCB: DNCB-treated control group, DNCB-chamaejasmine: DNCB and 0.5% chamaejasmine-treated group, DNCB-Elidel: DNCB and 1% pimecrolimus cream-treated group.

The major biochemical characteristics of AD are increased serum levels of IgE and IL-4. Serum IgE levels were significantly higher in DNCB controls than in untreated controls. Treatment with 0.5% **7** resulted in a 38% decrease in serum IgE concentration *versus* DNCB controls (Figure 6a). Mean total serum IL-4 level (42.5 pg/mL) was also higher in DNCB controls than in untreated controls (17.4 pg/mL). Mean total serum IL-4 levels in the DNCB-chamaejasmine group (25.2 pg/mL) were markedly lower than in DNCB controls (Figure 6b). Pimecrolimus cream decreased serum IgE levels by 43% (Figure 6a) and serum IL-4 levels by 27% (Figure 6b) *versus* those in DNCB controls.

### 3.4. Chamaejasmine (7) from W. dolichantha Recovered Skin Barrier Function in DNCB-Induced Atopic Mice 

**Figure 7:**
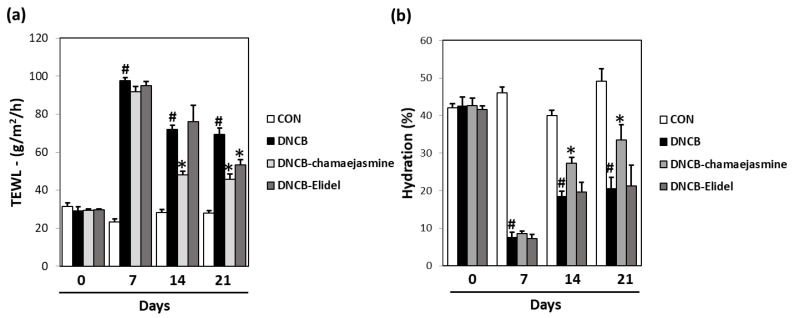
Effects of chamaejasmine on skin barrier function in DNCB-sensitized SKH-1 hairless mice. (**a**) Transepidermal water loss (TEWL) and (**b**) skin hydration values Results are expressed as the means ± SEMs (*n* = 7) of two independent experiments. ^#^ *P* < 0.05 vs. the CON group; * *P* < 0.05 vs. the DNCB group. EtOH extract of *Wikstroemia dolichantha*, CON: vehicle control group, DNCB: DNCB-treated control group, DNCB-chamaejasmine: DNCB and 0.5% chamaejasmine-treated group, DNCB-Elidel: DNCB and 1% pimecrolimus cream-treated group.

A significant increase in dorsal skin TEWL was observed in DNCB controls on the last day of the experiment. In contrast, treatment with 0.5% **7** (43.5 g/m^2^h) and 0.5% pimecrolimus cream (46.7 g/m^2^h) markedly reduced TEWL as compared with DNCB controls (68.9 g/m^2^h) (Figure 7a). Furthermore, a significant decrease in skin hydration was observed in DNCB controls during the 21-day experimental period, but skin hydration was significantly higher in the DNCB-chamaejasmine group (45% increase) than in DNCB controls (Figure 7b).

## 4. Discussion

Flavonoids are a large family of plant secondary metabolites, are found in all fruits and vegetables [21], and are primarily responsible for the characteristic red and blue colors of flowers and berries [22]. Over 4,000 flavonoids have been identified to date and are classified as flavonols, flavones, flavanones, catechins, anthocyanidins, isoflavones, dihydroflavonols, or chalcones [23]. The bioactivities of flavonoids are related to their chemical structures and functionalities [24,25]. Flavonoids are known to have health-promoting effects, which have been largely attributed to their antioxidant, anti-inflammatory, and chelating properties [25,26,27]. However, experimental evidence regarding their anti-allergic effectiveness is scarce and limited to a few individual flavonoids under highly specific experimental conditions.

Our in vitro studies on the immunological and anti-inflammatory activities of WDE and its *n*-hexane (60.3 g), EtOAc (39.9 g), *n*-BuOH (68.0 g), and water (206 g) fractions revealed the EtOAc soluble fraction of WDE inhibited β-hexosaminidase release from RBL-2H3 cells. Ten flavonoids, that is, padmatin (**1**), aromadendrin (**2**), apigenin (**3**), wikstaiwanone C (**4**), taxifolin (**5**), neochamaejasmine B (**6**), chamaejasmine (**7**), naringenin (**8**), afzelechin (**9**), and catechin (**10**), were obtained from the active EtOAc soluble fraction by silica gel column chromatography, Sephadex LH-20 column chromatography, and RP HPLC. The inhibitory effects of all ten compounds were tested against the release of β-hexosaminidase induced by dinitrophenylated bovine serum albumin (DNP-BSA) from RBL-2H3 cells, using ketotifen as a reference. Of the ten isolates, **2**, **3**, **4**, **5**, **6** and **7** strongly inhibited β-hexosaminidase release, and interestingly, all biflavonoids tested (wikstaiwanone C, neochamaejasmine B, and chamaejasmine) inhibited mouse β-hexosaminidase. Interestingly, chamaejasmine (**7**) had stronger β-hexosaminidase activity than wikstaiwanone C (**4**), which was attributed to the presence of one methoxy group in the B ring of chamaejasmine (**7**), which concurs with previous suggestions that the presence of a methoxy group in bioflavonoids might substantially increase anti-inflammatory activity [28,29]. In the present study, chamaejasmine (**7**) showed excellent anti-inflammatory activity, and thus, its anti-AD properties were studied in the DNCB-sensitized SKH-1 hairless mouse model.

Hapten-induced mouse models of contact dermatitis have been commonly used to investigate anti-AD properties, such as those of oxazolone, DNCB, and 1, 3, 5-trinitrochlorobenzene (TNCB). DNCB is considered to be useful for inducing sensitization due to its availability and reproducibility [30,31]. Topical administration of 0.5% chamaejasmine (**7**) for two weeks markedly attenuated DNCB-induced AD-like skin lesions, which included hyperkeratosis, epidermal thickening, and mast cell infiltration, in our murine model. Moisturizers increase skin hydration and improve skin barrier function, thereby serving as an important first-line therapeutic option for patients with AD increasing skin hydration [32]. Chamaejasmine (**7**) also protected skin barrier functions by increasing TWEL and skin hydration as a moisturizer and suppressing inflammatory cytokine IL-4 production versus DNCB controls. Reduced skin barrier function and inflammation have been reported to be associated as impaired skin barrier function facilitates allergen entry and thus inflammation and inflammation, which in turn, may comprise barrier functions [33,34]. Accordingly, our observations suggest chamaejasmine (**7**) treatment may inhibit T_H_2-dominated inflammatory response. In addition, chamaejasmine (**7**) suppressed allergic inflammation by strongly inhibiting DNCB-induced increases in serum IgE and IL-4 levels. Application of chamaejasmine (**7**) markedly suppressed the expression level of IL-4 *in vivo*. It is suggested a possible mechanism that inhibition of IL-4 prevents the differentiation of Th0 to Th2 cells in lymph nodes, the IgE class switch of B cells caused by IL-4 secreted from Th2 cells, and the degranulation of mast cells by IgE, which mediate lowering allergic response. When Th2 cells migrate from lymph nodes to skin tissues, IL-4 increases the expression of proteases such as Kallikreins in keratinocytes of the epidermis, which weaken the binding between epidermal cells and skin barrier function. In addition, macrophage activates the STAT6 pathway downstream of IL-4, leading to polarization to M2. Treatment of chamaejasmine (**7**) is expected to prevent the above symptoms by IL-4 and reduce the worsening of AD.

A considerable body of evidence suggests that plants flavonoids are health-promoting and disease-preventing dietary compounds [35]. However, the potential therapeutic use of flavonoids and flavonoid-rich extracts in infants and children remains a matter of debate [36]. Some well-known flavones and flavonols, such as, quercetin and kaempferol, have been especially well analyzed for their immunological effects [37]. Previous structure-activity studies with flavonoids have shown that flavones or flavonols were active in inhibiting degranulation of RBL-2H3 cells but flavanones, isoflavones, and catechins were not active [38,39]. In contrast to previous results on the flavanones, our present study suggests that flavanones (aromadendrin and taxifolin) and flavanone dimers (wikstaiwanone C, neochamaejasmine B, and chamaejasmine) may be useful inhibitors of β-hexosaminidase. Further studies on whole cell systems and on other flavonoids, especially flavanones, will be reported in due course.

Six flavonoids isolated from *W. dolichantha* significantly reduced IgE-induced β-hexosaminidase release from RBL-2H3 cells, and chamaejasmine (**7**) was found to be the most active. Topical chamaejasmine (**7**) application impressively reduced erythema, edema, erosion, dryness, and lichenification, in our SKH-1 mouse model by suppressing serum IgE and IL-4 levels. Collectively, these results suggest **7** be viewed as a novel anti-inflammatory and anti-atopic agent with promising therapeutic potential.
